# Visual and patient reported outcomes provided by a refractive multifocal intraocular lens based on continuous transitional focus

**DOI:** 10.1186/s40662-024-00408-y

**Published:** 2024-10-14

**Authors:** Jorge L. Alió, Antonio Martínez-Abad, Ramón Ruiz-Mesa, Hyo Myung Kim, Javier Mendicute, Filomena J. Ribeiro, Mike P. Holzer, Mario Cantó-Cerdán

**Affiliations:** 1 Vissum Miranza, Avda de Denia s/n Edificio Vissum, 03016 Alicante, Spain; 2 Division of Ophthalmology, Universidad Miguel Hernández, Alicante, Spain; 3 Oftalvist CIO Jerez, Cádiz, Spain; 4 Department of Ophthalmology, Korea University Anam Hospital, Seoul, Republic of Korea; 5 Department of Ophthalmology, Hospital Universitario Donostia, San Sebastián, Spain; 6 Departamento de Oftalmologia do Hospital da Luz Lisboa, Universidade de Lisboa, Lisboa, Portugal; 7 Department of Ophthalmology, International Vision Correction Research Centre (IVCRC), University of Heidelberg, Heidelberg, Germany; 8 Avda de Denia s/n Edificio Vissum, Alicante, 03016 Spain

## Abstract

**Purpose:**

To analyze the quality of vision of patients implanted bilaterally with the multifocal Precizon Presbyopic intraocular lens (IOL), as well as to evaluate the visual performance provided by the lens.

**Setting:**

Vissum Miranza Alicante.

**Design:**

Prospective multicenter study.

**Methods:**

56 patients (mean age 65.0 ± 8.7 years old) underwent bilateral implantation with multifocal Precizon Presbyopic IOL. The quality of vision was assessed by a quality of vision questionnaire at 6 months after the implantation procedure, a complete eye examination was also performed including visual and refractive measurements, defocus curve and contrast sensitivity assessment. Visual and refractive variables were compared in preoperative, 3-month postoperative and 6-month postoperative visits by Wilcoxon test.

**Results:**

The quality of vision analysis showed the absence of severe glare and severe haloes in all evaluated patients. Likewise, non-symptoms of glare, haloes and starbursts were seen in 75%, 68%, and 55% of subjects, respectively. Efficacy and safety index was 1.26 and 1.42, respectively. The 6-month postoperative binocular uncorrected distance visual acuity and near uncorrected visual acuity were 0.00 ± 0.09 and 0.20 ± 0.13 logMAR, respectively. Mean spherical equivalent was 0.29 ± 0.45 D.

**Conclusions:**

The Precizon Presbyopic NVA IOL (OPHTEC BV) provides a suitable quality of vision with a low rate of disturbance photic phenomena induction, as well as an excellent visual performance at main distances of sight accomplishing the visual demands of the majority of patients.

**Supplementary Information:**

The online version contains supplementary material available at 10.1186/s40662-024-00408-y.

## Background

Nowadays, the social demand for multifocal intraocular lenses (IOLs) has increased in tandem with more younger patients demanding a solution for their presbyopia and looking for spectacle independence either at the moment of cataract surgery or when a refractive lens exchange is indicated for refractive purposes [1, 2]. Over the last decade, multifocal designs composed of different technologies have increasingly developed and commercialized, demonstrating in current models an excellent visual acuity on various distances of sight, as well as in other visual functions such as contrast sensitivity or defocus curve [3–6]. However, patients report unsatisfactory outcomes concerning their visual quality such as uncomfortable photic phenomena despite having optimal visual acuity. It is commonly associated with the IOL optic design and neuroadaptation failures that eventually may lead to a new surgery with IOL exchange [3, 7–9].

The Precizon Presbyopic NVA (OPHTEC BV) multifocal IOL has been created with an innovative optical design based on its aspherical polisegmented refractive optic, forming a continuous transitional focus (CTF) (Figure S1). In previous reports, this lens has been reported to have very good clinical outcomes [10–17].

A suitable and patient-reported outcome is an excellent way to evaluate patient satisfaction, especially using validated tests such as the quality of vision (QoV) questionnaire to assess photic phenomena perceived by patients. It is an instrument designed to characterize the severity, frequency, and bothersome nature of 10 dysphotopic symptoms based on illustrated photographs. Particularly, the dysphotopic symptoms included in the questionnaire are the following: glare, haloes, starbursts, hazy vision, blurred vision, distortion, double vision, fluctuations in vision, focusing difficulties, and difficulty judging distance or depth perception [18].

The aim of this multicenter prospective study was to analyze the visual and patient-reported outcomes of patients implanted bilaterally with the multifocal Precizon Presbyopic IOL and to evaluate the visual performance provided by the lens during the follow-up postoperative period.

## Methods

### Study design

This is a prospective and multicenter study conducted at six different sites: Vissum Miranza Alicante (Spain), Augenzentrum Prof. Dr. Holzer & Prof. Dr. Rabsilber (Germany), Oftalvist CIO Jerez (Spain), Hospital Universitario Donostia (Spain), Hospital da Luz (Portugal) and Korea University Anam Hospital (Republic of Korea).

### Patients

Patients who underwent bilateral implantation of the Precizon Presbyopic multifocal IOL, presented with significant cataract or presbyopia associated with refractive lens dysfunction (RLD), and wished to be spectacle-independent for near and far distance. Exclusion criteria were the presence of any other ocular comorbidity besides cataract (anterior segment anomalies, glaucoma, corneal dystrophies, retinal disorders, or neuro-ophthalmic disease), amblyopia, previous ocular surgeries, acute or chronic systemic diseases potentially affecting visual abilities, and preoperative corneal astigmatism higher than 1.0 D. All patients who met the inclusion criteria and were motivated to participate in the study agreed to participate and signed a written informed consent form. The study adhered to the tenets of the Declaration of Helsinki and was approved by Ethics Committee for Drug Research of Cádiz from Spain (AP01000740).

### Intraocular lens

The Precizon Presbyopic IOL NVA model 570 (OPTHEC BV) is a one-piece IOL made of a hybrid material hydrophilic/hydrophobic acrylic material with ultraviolet filtering HEMA/EOEMA copolymer, and a refractive index of 1.46. The size of the clear optic diameter is 6.0 mm, with an overall diameter of 12.5 mm.

A multi-zonal refractive design allows the lens to maintain the light distribution and exposure on the foci regardless of the tilt or decentering of the lens. This IOL provides the ability for a transition in focus between 11 distinct segments (five for distance and six for near vision) with the central segment dedicated for distance vision. The rotated segments have a width of 0.60 mm, and these segments are distributed in such a way that decentration or pupil size has a minimal effect on the ratio between near and far correction [11].

The IOL optic is designed to provide a CTF divided into three concentric sectors: the central sector, of higher diameter, is dedicated to distance correction; two peripheral sectors present a bimodal (50%–50%) distribution of distance and near correction, and this distribution changes along four segments in each sector. This new optic design with an anterior surface with multiple segments for far and near achieves a soft transition from far to near focus. This transition offers a constant progressive focus between the two sharp focal points to facilitate a sharp image on the retina while delivering a good intermediate vision [19].

This IOL is provided with an optic power range between + 1.0 and + 35.0 D (0.5 D increments) and has an addition of + 2.75 D for near focus. A toric version of the lens is now also available. Standard phacoemulsification using a 2.2-mm clear corneal incision was performed in every case by all investigators with no significant variations in the surgical technique.

### Pre- and postoperative examination protocol

All patients underwent preoperative complete eye examination, including uncorrected distance visual acuity (UDVA), corrected distance visual acuity (CDVA) [both monocularly and binocularly measured with the Early Treatment Diabetic Retinopathy Study (ETDRS) LogMAR Charts at 4 m distance], subjective refraction, binocular contrast sensitivity in photopic and mesopic conditions in the presence and absence of glare (CSV-1000, Vector Vision), biometry (IOL Master, Zeiss), pupillometry and corneal topography (Sirius, Costruzione Strumenti Oftalmici), slit-lamp and fundus examination. After 6 months postoperative, ocular and visual examinations included: monocular and binocular UDVA, CDVA, uncorrected near visual acuity (UNVA) and distance corrected near visual acuity (DCNVA) (ETDRS reading chart calibrated at 40 cm); subjective refraction, contrast sensitivity in the same condition to preoperative evaluation, binocular defocus curve from − 5.00 to + 1.50 D following the conventional procedure [20–22]; patient reported outcomes (PROMs) were evaluated with the QoV questionnaire [18]. Main outcome measures were differences on efficacy (percentage of eyes that showed equal or better UDVA compared with preoperative CDVA) and safety [percentage of eyes that lost lines (Snellen) of CDVA after the primary procedure compared with preoperative CDVA]. During the follow-up of this study, YAG laser capsulotomy was not performed in any of the cases. All measurements were performed by optometrists and ophthalmologists certified in Good Clinical Practice.

### Assessment

The primary measurement assessed in this study was photic phenomena symptoms using the 10-item QoV questionnaire score. It is a validated questionnaire presenting 10 dysphotopic symptoms illustrated by photographs. Patients have to score each item (0, 1, 2 and 3) according to the frequency (never, occasionally, quite often, very often), the severity (not at all, mild, moderate and severe, respectively), and bothersome perceptions [6]. Incidence data of complaining levels for each dysphotopic symptom was assessed and described, as well as the representation of bothersome graphs represented by histogram charts for the most common symptoms. Moreover, the value of the responses to the questionnaire is presented in the Raw Score.

Additional variables requiring complete analysis were the visual functions mentioned in the examination protocol. Visual acuities at the most important distance of sight and all parameters involved in ocular refraction were reported and compared between the preoperative visit and several postoperative visits. Monocular and binocular evaluations were established to assess the visual performance provided by the lens (monocularly) and the visual abilities obtained by the patient (binocularly). Binocular contrast sensibility and binocular defocus curves were characterized 6 months postoperatively in all patients.

### Statistical analysis

The statistical analysis was performed with the SPSS software for Windows (IBM SPSS Statistics, version 26.0). The non-normality of the study sample was confirmed using the Kolmogorov–Smirnov test; non-parametric tests were needed. Wilcoxon test was used to assess the difference between the preoperative and postoperative outcomes, and Spearman’s rho correlation coefficient test was used for ordinal data. Differences were considered statistically significant when the *P* value was less than 0.05.

Visual acuity was measured using the logMAR scale. The standardized graphs and terms for refractive surgery outcomes were used [23].

## Results

### Subjective refractive and visual outcomes

A total of 112 eyes of 56 patients (89.7% cataract, 10.3% crystalline lens dysfunction) were examined 6 months postoperatively. Table 1 shows monocular corrected and uncorrected visual acuities for the main distances of sight, as well as the refraction data preoperatively and at different stages of postoperative follow-up. Refractive improvement was observed after the surgery and mean values lower than 0.50 D in terms of sphere, cylinder, and spherical equivalent (SE). UDVA and CDVA improved after surgery at 3 and 6 months (*P* = 0.022 and *P* = 0.012, respectively). The mean outcomes at 6 months of UNVA and DCNVA were 0.28 ± 0.16 and 0.24 ± 0.14 logMAR respectively, with a significant improvement between the third and sixth months (*P* = 0.009 and *P* = 0.004, respectively). At 3 months, the mean corrected near visual acuity (CNVA) was 0.12 ± 0.09 logMAR and mean uncorrected intermediate visual acuity (UIVA) was 0.22 ± 0.12 logMAR.

**Table 1:** Preoperative and postoperative visual acuities and refractive outcomes at 3 and 6 months for 112 eyes

Parameter	Preop	3 M postop	6 M postop	*P* value pre–3 M	*P* value pre–6 M	*P* value 3 M–6 M
*Monocular*						
Sphere (D)						
Mean ± SD	0.84 ± 2.13	0.28 ± 0.46	0.29 ± 0.45	0.001	0.001	0.983
Range	−5.50, 5.25	−1.00, 1.50	−0.50, 1.50			
Cylinder (D)						
Mean ± SD	−0.66 ± 0.60	−0.29 ± 0.40	−0.29 ± 0.39	< 0.001	< 0.001	0.876
Range	−3.00, 0.00	−1.25, 0.00	−1.25, 0.00			
SE (D)						
Mean ± SD	0.51 ± 2.15	0.14 ± 0.41	0.14 ± 0.39	0.013	0.008	0.993
Range	−5.50, 4.75	−1.25, 1.25	−0.63, 1.38			
UDVA (logMAR)						
Mean ± SD	0.54 ± 031	0.07 ± 0.12	0.06 ± 0.12	< 0.001	< 0.001	0.022
Range	−0.06, 1.30	−0.16, 0.44	−0.16, 0.48			
CDVA (logMAR)						
Mean ± SD	0.19 ± 0.23	0.01 ± 0.08	0.00 ± 0.08	< 0.001	< 0 0.001	0.012
Range	−0.16, 1.30	−0.16, 0.30	−0.16, 0.30			
UNVA (logMAR)						
Mean ± SD	–	0.30 ± 0.13	0.28 ± 0.16	–	–	0.007
Range	–	0.06, 0.70	0.00, 0.78			
DCNVA (logMAR)						
Mean ± SD	–	0.27 ± 0.12	0.24 ± 0.14	–		0.004
Range	–	0.04, 0.56	0.00, 0.70			
CNVA (logMAR)						
Mean ± SD	–	0.12 ± 0.09	–	–	–	–
Range	–	0.00, 0.56	–			
UIVA (logMAR)						
Mean ± SD	–	0.22 ± 0.12	–	–	–	–
Range	–	0.04, 0.60	–			
*Binocular*						
UDVA (logMAR)						
Mean ± SD	0.38 ± 0.28	−0.01 ± 0.07	0.00 ± 0.09	< 0.001	< 0.001	0.567
Range	−0.06, 1.00	−0.16, 0.12	−0.22, 0.30			
CDVA (logMAR)						
Mean ± SD	0.09 ± 0.14	−0.03 ± 0.05	−0.04 ± 0.08	< 0.001	< 0.001	0.109
Range	−0.18, 0.40	−0.16, 0.10	−0.24, 0.18			
UNVA (logMAR)						
Mean ± SD	–	0.20 ± 0.11	0.20 ± 0.13	–	–	0.154
Range	–	0.00, 0.50	−0.04, 0.50			
DCNVA (logMAR)						
Mean ± SD	–	0.20 ± 0.11	0.17 ± 0.12	–	–	0.001
Range	–	0.00, 0.50	−0.04, 0.40			
CNVA (logMAR)						
Mean ± SD	–	0.08 ± 0.07	–	–	–	–
Range	–	0.00, 0.30	–			
UIVA (logMAR)						
Mean ± SD	–	0.14 ± 0.10	–	–	–	–
Range	–	0.00, 0.50	–	–	–	–

*D* = diopters; *M* = month; *UDVA* = uncorrected distance visual acuity; *CDVA* = corrected distance visual acuity; *UNVA* = uncorrected near visual acuity; *DCNVA* = distance corrected near visual acuity; *CNVA* = corrected near visual acuity; *UIVA* = uncorrected intermediate visual acuity

Table 1 also shows the binocular corrected and uncorrected visual acuity for the same distances. The outcomes provided by this IOL confirmed a postoperative improvement in UDVA and CDVA (both *P* < 0.001), showing no changes between the third and sixth months (*P* = 0.567 and *P* = 0.109, respectively). Mean binocular UNVA, DCNVA, CNVA and UIVA were lower than 0.30 logMAR and considerably better than monocular measurements.

### Efficacy and safety

**Fig. 1:**
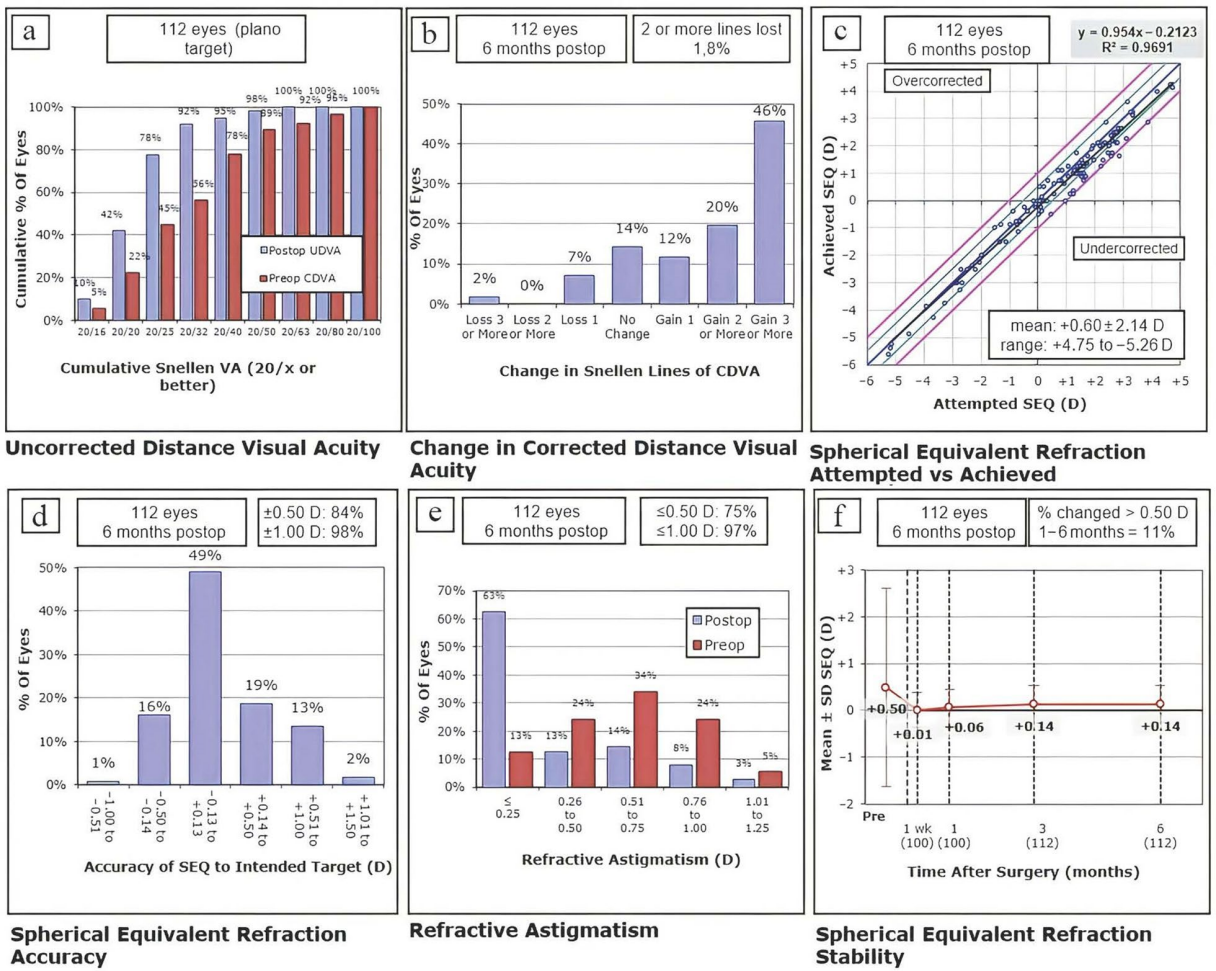
Long term outcomes (6 months). **a** Comparison of last visit postoperative uncorrected distance visual acuity (UDVA) and preoperative corrected distance visual acuity (CDVA) (efficacy). **b** Changes in lines of CDVA last visit after surgery (safety). **c** Intended versus achieved correction (spherical equivalent refraction) last visit after surgery. **d** Distribution of postoperative spherical equivalent (predictability) last visit after surgery. **e** Distribution of preoperative and postoperative astigmatism. **f** Stability of the manifest refraction over time

Efficacy index was 1.26 at 6 months. Eighty-seven eyes (78%) had a UDVA ≥ 20/25 (Fig. 1a). Safety index was 1.42 at 6 months. CDVA remained the same or improved in 102 eyes (91%) 6 months after surgery (Fig. 1b).

### Predictability

At 6 months, 49% of eyes had an SE within ± 0.13 D, 84% within ± 0.50 D, and 98% within ± 1.00 D (Fig. 1d). The scatterplot of the attempted versus achieved SE correction at the sixth postoperative month is shown in Fig. 1c.

### Astigmatism analysis

Six months after surgery, 84 eyes (75%) had astigmatism ≤ 0.50 D and 109 eyes (97%) had astigmatism ≤ 1.00 D (Fig. 1e).

### Stability

No statistically significant differences were detected between the third and sixth postoperative months in any of the refractive parameters (Table 1). SE changed over 0.50 D in 11% of the eyes (Fig. 1f).

### Defocus curve

**Fig. 2:**
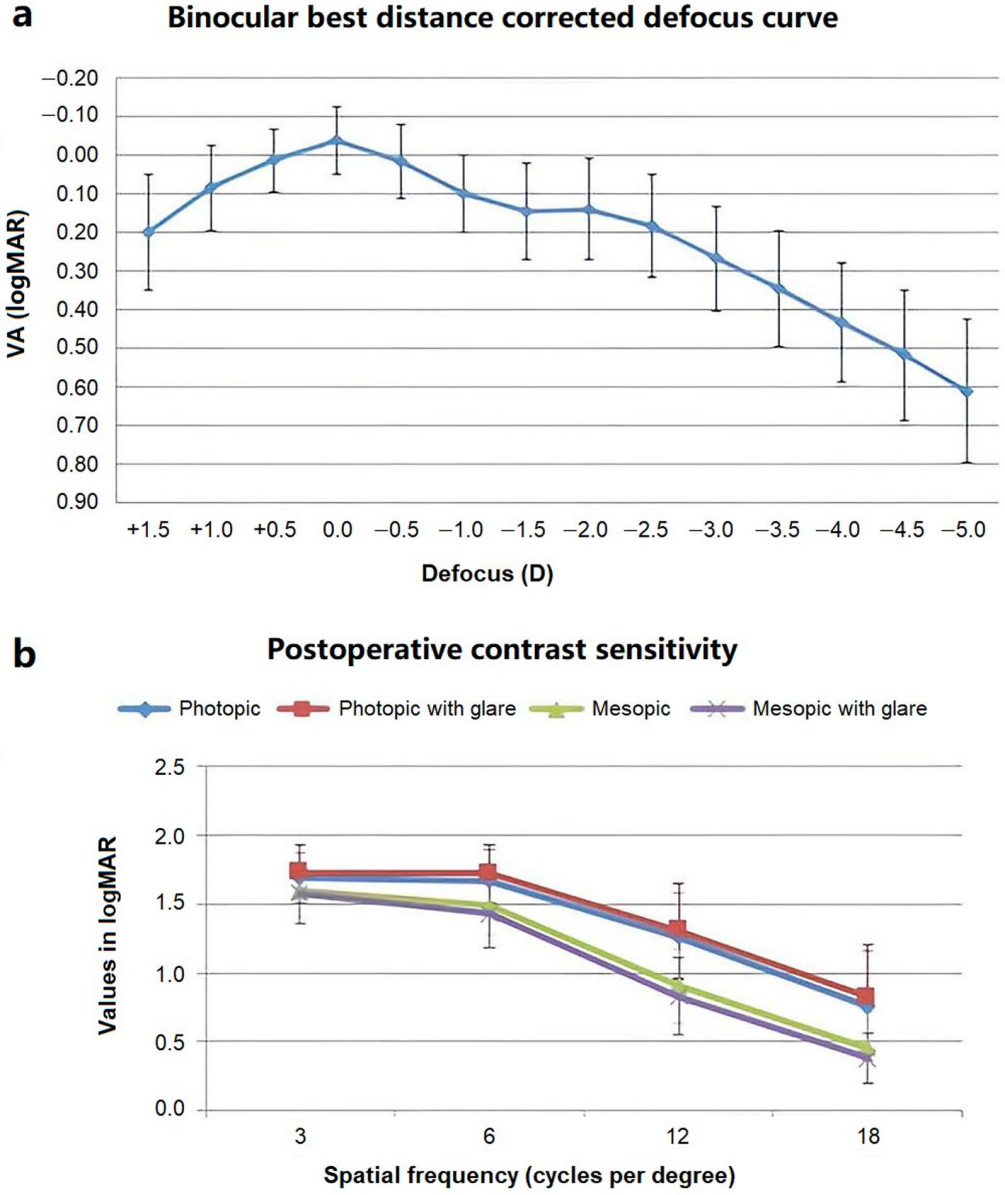
Results at 6 months. **a** Binocular defocus curve. **b** Contrast sensitivity measured with CSV-1000 test

Figure 2a shows the binocular defocus curve, reporting visual acuities between 0.10 and 0.20 logMAR for a range of defocus between − 1.50 and − 2.50 D. This is consistent with intermediate and near vision, demonstrating suitable visual function for a wide range of distances.

### Contrast sensitivity

Figure 2b shows the graphical representation outcomes of contrast sensitivity in photopic and mesopic conditions and measurement with and without glare in binocular vision at 3 months after implantation. It can be observed that this multifocal IOL provides a suitable contrast sensitivity value (in log units) in photopic conditions of lighting and in the absence of glare for different spatial frequencies, 3 cycles per degree (A), 6 cycles per degree (B), 12 cycles per degree (C) and 18 cycles per degree (D) (A: 1.69 ± 0.18; B: 1.67 ± 0.23; C: 1.26 ± 0.31; D: 0.76 ± 0.39). It was slightly reduced in mesopic lighting conditions and in the presence of glare (A: 1.59 ± 0.19; B: 1.49 ± 0.22; C: 0.90 ± 0.27; D: 0.38 ± 0.27). Values for photopic with glare (A: 1.73 ± 0.20; B: 1.72 ± 0.21; C: 1.31 ± 0.34; D: 0.83 ± 0.38) and mesopic with glare (A: 1.58 ± 0.22; B: 1.43 ± 0.25; C: 0.83 ± 0.28; D: 0.38 ± 0.18) also were represented.

### Patient reported outcomes (PROMs): Quality of Vision

**Fig. 3:**
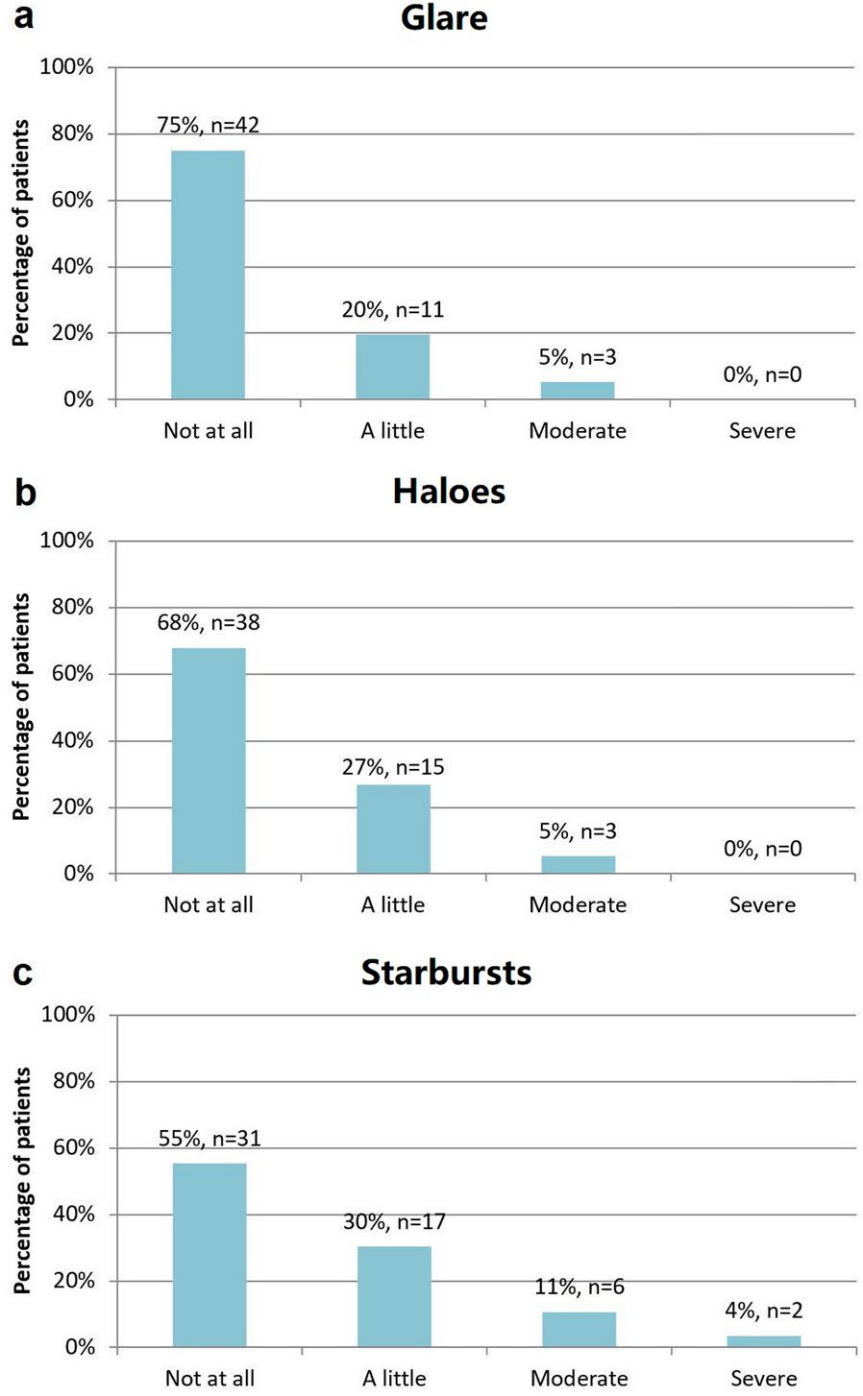
Outcomes of quality of vision questionnaire at 6 months postoperatively **a** Glare; **b** Haloes; **c** Starbursts

Table 2 shows the mean QoV score for which each item was rated from 0 to 3 depending on the frequency and/or severity of each dysphotopic symptom. Figure 3 summarizes the severity and frequency of the most common dysphotopic symptoms (glare, haloes, and starbursts) for only one item. It shows the practically non-existent rate of very uncomfortable symptoms because no patient had severe haloes or glare, and only 4% of patients complained of severe starbursts. In addition, the majority of patients did not perceive any of these symptoms, and in the case of perceiving it, they were of occasional character or mild severity.

**Table 2:** Mean quality of vision (QoV) scoring corresponding to each dysphotopic symptom at 6 months for 56 eyes

Symptom	How often do you experience it?	How severe is it?	How bothersome is it?
Glare	0.55 ± 0.87	0.57 ± 0.89	0.30 ± 0.57
Haloes	0.66 ± 0.88	0.61 ± 0.78	0.38 ± 0.59
Starbursts	1.02 ± 1.10	0.96 ± 1.01	0.63 ± 0.82
Hazy vision	0.32 ± 0.64	0.30 ± 0.63	0.20 ± 0.48
Blurred vision	0.30 ± 0.60	0.32 ± 0.66	0.21 ± 0.49
Distortion	0.21 ± 0.65	0.21 ± 0.65	0.20 ± 0.64
Double vision	0.20 ± 0.70	0.21 ± 0.73	0.13 ± 0.51
Fluctuation in vision	0.39 ± 0.76	0.46 ± 0.89	0.39 ± 0.85
Focusing difficulties	0.52 ± 0.74	0.52 ± 0.76	0.38 ± 0.65
Difficulty judging distance or depth perception	0.18 ± 0.58	0.20 ± 0.64	0.16 ± 0.50

Grading scale: 0 = never or not all; 1 = occasionally or mild; 2 = quite often or moderate; 3 = very often or severe

Regarding the rest of the symptoms included in the questionnaire, the outcomes were even more satisfactory, not obtaining any patient with severe hazy vision, blurred vision, or focusing difficulties, and obtaining a very low rate of presenting distortion, double vision, and fluctuation in vision with severe characteristics (less than 5%). The complete absence of dysphotopic symptoms was observed in a high percentage of implanted individuals: 84% of hazy vision, 82% of blurred vision, 89% of distortion, 93% of double vision, 79% of fluctuations in vision, 71% of focusing difficulties, and 89% of difficulty judging distance or depth perception.

## Discussion

The development and generalization of multifocal IOLs have led to significant progress in the surgical correction of aphakia. Providing good distance, intermediate, and near vision is crucial to determine the performance of any lens because of the greater visual demands in the current society and the desire for spectacle independence [1, 2]. In addition, the study confirmed the good visual and refractive outcomes of the Precizon Presbyopic lens in a multicenter study, and subjects reported a good quality of vision with the implantation of this lens, with a low rate of disturbance photic phenomena induction [10, 17]. Visual outcomes obtained in this study demonstrated an excellent UDVA and acceptable UNVA and UIVA considering monocular conditions. Monocular evaluation allows the evaluation of the isolated performance provided by the lens, but they are commonly implanted bilaterally, and these data are more suitable for evaluating the visual abilities perceived by the patient and, especially, the PROMs provided by a new lens design.

In this study, the refractive multifocal lens design offered excellent outcomes for binocular UDVA and a relevant improvement in UNVA and UIVA. These results were reproduced in this multicenter study, the ones reported in a monocenter pilot study performed by Alio et al. using the same lens [10]. Such outcomes are confirmed in this multicenter investigation that confirms the visual outcomes using an extended sample and the participation of several centers. Similarly, Royo et al. [11] developed an independent study with identical lens and obtained a mean binocular UDVA of 0.01 ± 0.03 logMAR, UNVA of 0.02 ± 0.04 logMAR, and UIVA of 0.17 ± 0.04 logMAR. They obtained similar results for far and intermediate distances, but better near visual acuity in comparison with the present study. This could be explained by the slightly more myopic state they obtained (mean SE of − 0.50 D vs. 0.14D) and by the sample variability, which was minimized in our study due to the multicenter design. In any case, the postoperative results were considerably better than the preoperative level at all distances of sight, demonstrating excellent efficacy in terms of visual acuity. In addition, the defocus curve showed acceptable results for moderate and high negative defocus, which is consistent with the good visual acuity measured at 80 cm and 40 cm. Subsequent studies have confirmed the good visual and refractive performance of this IOL [12–17].

The comparison of visual data provided by this lens based on CTF versus other multifocal designs reveals a very similar UDVA [between − 0.10 and 0.10 logMAR] and even better results than previous reports with some diffractive and refractive commercialized IOLs: logMAR VA 0.18 with FineVision IOL [24], 0.17 with Lentis MPlus [25], 0.07 with Acrysoft Vivity [26], − 0.02 with SBL [27], − 3 or 0.12 with Tecnis ZMA00 [28]. Mean UIVA was better than 0.30 logMAR monocularly and better than 0.20 logMAR binocularly, which is consistent with acceptable intermediate vision according to results achieved in the 96% of IOLs destined to compensate intermediate vision [3]. Mean UNVA was also better than 0.30 logMAR indicating a similar performance for near activities compared to other multifocal designs and better results than low addition multifocal designs [25], and some extended depth-of-focus IOLs [29].

The absence of postoperative residual refractive errors reflects the refractive predictability of this lens, which may be due to the IOL power calculation. Likewise, longitudinal analysis of the visual data showed no statistically and/or clinically significant differences between the different postoperative visits, indicating suitable stability at 6 months.

Regarding contrast sensitivity, it used to be decreased in the majority of diffractive IOLs compared to monofocal designs due to the light distribution in several foci. It was measured under several light conditions, considering the absence and presence of a glare. The results under photopic conditions were acceptable, even in the presence of glare, and very similar to other IOLs designs. In mesopic conditions, contrast sensitivity was slightly reduced at all spatial frequencies, which is consistent with previous evidence with other lenses [30].

Concerning PROMs, the subjective quality of vision, which is probably the most important factor for good patient satisfaction, was evaluated from the dysphotopic symptoms provided by the validated QoV questionnaire which is a main strength compared to Royo et al.’s investigation [11]. They reported the percentage of disturbing haloes (9.7%), glare (6.5%) and starbursts (0.0%) but did not use a validated questionnaire and did not classify stage disturbances, which may be crucial for a complete assessment. The QoV scoring comparison obtained in the current study demonstrated similar or better outcomes than other refractive asymmetric multifocal designs showing a lower overall scoring of glare (0.30) and haloes (0.38) than SBL-3 IOL (Lenstec, Inc.) with a mean scoring of 0.58 in glare and 0.43 in haloes; and Lentis MPlus LS-312 MF 30 (Oculentis GmbH) with a mean scoring of 0.50 and 0.43, respectively. Regarding scoring of starburst perception (0.63) it was very similar compared to mentioned asymmetric lenses (0.63–0.75) [27]. Likewise, the QoV comparison of this lens versus other diffractive multifocal designs reveals very close rates of glare and haloes, showing similar percentages of patients completely absent of glare [75.0% in our study, 75.0% with AT Lisa 809 M (Carl Zeiss Meditec) [31], 60.0% with Rayone (Rayner) [32], and 33.3% with FineVision (PhysIOL) [32]] and haloes [68.0% in our study, 56.0% with AT Lisa 809M [33], 60.0% with Rayone [32], and 46.7% with FineVision] [32], as well as lower rates of very disturbing haloes and glare [0.0% in our study, and 4.8% 6.0% with AT Lisa 809 [31]. The current outcomes confirm the low rate of disturbance photic phenomena encountered with the CTF IOL, which is similar to other commercialized multifocal lenses in absolute terms. It also differs in the predominant dysphotopic symptom which is the starburst perception and not haloes or glare perception; the predominant symptom in multifocal lenses and extended-depth-of-focus lenses [33]. The misalignment tolerance and use of segments instead of concentric rings reduce photic phenomena, helping patients adapt more naturally to their new vision.

The main strength of this study is that it is the first to evaluate PROMs and visual outcomes obtained by a CTF IOL using a validated questionnaire in a multicenter sample, which provides evidence regarding the visual symptoms perceived by patients implanted bilaterally by this lens. However, this study has some limitations. In this case, sampling was performed consecutively, and all patients implanted with the new lens were included. As this was not a random sample, patient selection bias could have occurred, and to minimize error, the inclusion and exclusion criteria were strictly followed. Another limitation is that pupillary diameter measurements were not reported although pupils were assessed clinically in all cases for implantation, and cases with abnormal pupils were excluded. These results were not reported in the statistical analysis and, therefore, cannot be compared. Finally, another limitation is that part of the study was based on subjective questionnaires answered by the patients. Hence, this study may have had a certain degree of patient expectation bias. In addition, the questionnaire scores are presented in the Raw Score and not in Logit score.

## Conclusions

In conclusion, the bilateral implantation of a CTF IOL Precizon Presbyopic refractive multifocal IOL offers a suitable quality of vision with a low rate of disturbance photic phenomenon induction. Likewise, it provides excellent visual performance at the main distances of sight, accomplishing the visual demands for the majority of patients.

### What was known?

- Precizon Presbyopic provides better optical quality and reduces photic phenomena, as reported in previous monocenter studies, with very good clinical outcome.

What does this paper add?

- This multicenter study confirms the good visual and refractive outcomes of the Precizon Presbyopic lens.
- Subjects reported a good quality of vision with the implantation of this lens, with a low rate of disturbance photic phenomena induction.

## Supplementary Information

Supplementary Material 1: Figure S1. Optical design representation on the Precizon Presbyopic NVA intraocular lens (Reprinted from Alió et al. [10]).

## Data Availability

The datasets used and/or analyzed during the current study are available from the corresponding author on reasonable request.
