# Supplementary figures and images for: Visual and patient reported outcomes provided by a refractive multifocal intraocular lens based on continuous transitional focus
Source: Eye Vis (Lond). 2024 Oct 14;11:41. doi: 10.1186/s40662-024-00408-y (PMC11479541; doi:10.1186/s40662-024-00408-y)

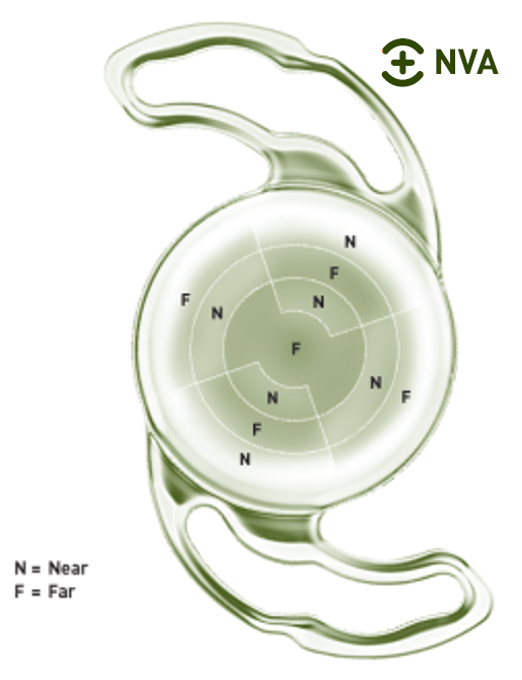

Supplement: Supplementary file 1 — Supplementary Material 1: Figure S1. Optical design representation on the Precizon Presbyopic NVA intraocular lens (Reprinted from Alió et al. [10]). [file 40662_2024_408_MOESM1_ESM.tif]
